# Real-Time Hand Posture Recognition for Human-Robot Interaction Tasks

**DOI:** 10.3390/s16010036

**Published:** 2016-01-04

**Authors:** Uriel Haile Hernandez-Belmonte, Victor Ayala-Ramirez

**Affiliations:** Universidad de Guanajuato DICIS, Carr. Salamanca-Valle Km. 3.5 + 1.8, Palo Blanco, Salamanca, C.P. 36885, Mexico; hailehb@laviria.org

**Keywords:** human-robot interaction, vision-based hand posture recognition, full image evaluation, AdaBoost, bootstrapping

## Abstract

In this work, we present a multiclass hand posture classifier useful for human-robot interaction tasks. The proposed system is based exclusively on visual sensors, and it achieves a real-time performance, whilst detecting and recognizing an alphabet of four hand postures. The proposed approach is based on the real-time deformable detector, a boosting trained classifier. We describe a methodology to design the ensemble of real-time deformable detectors (one for each hand posture that can be classified). Given the lack of standard procedures for performance evaluation, we also propose the use of full image evaluation for this purpose. Such an evaluation methodology provides us with a more realistic estimation of the performance of the method. We have measured the performance of the proposed system and compared it to the one obtained by using only the sampled window approach. We present detailed results of such tests using a benchmark dataset. Our results show that the system can operate in real time at about a 10-fps frame rate.

## 1. Introduction

Human-robot interaction (HRI) tasks are needed to enable humans and robots to perform tasks in a cooperative way. For example, Burger *et al.* [1] teach a robot how to perform an interactive manipulation task. Another example comes from the work of Muhlig *et al.* [2], where a robot is taught a kinematic sequence that it can reproduce in new situations. Kim, Sim and Yang [3] show how to command a cleaning robot to move to a specific place by using hand postures. The list is not exhaustive, but tries to point out some representative examples of HRI.

There are also different ways to interact with robots. The human can use different parts of his or her body to perform an interaction. The hand is the body part most used to make gestures [4] and to interact between humans. Furthermore, interaction by using hand gestures is one of the most intuitive ways to interact with a robot in conjunction with voice command. However, voice commands can be perturbed in noisy environments. Because of this, hand gesture recognition is a core element to develop an HRI system. Erol *et al.* [5] point out that the computer vision hand recognition problem poses several challenges and that it is far from being completely solved. The hand recognition system needs to be fast enough to recognize hand gestures and to work under different outdoor and indoor scenarios. We propose then to address this problem using an ensemble of hand detectors, based on the real-time deformable detector (RTDD).

A realistic performance evaluation of the hand posture detection and classification method is also needed to assess if it is useful for a given application. In this paper, we propose to use a performance evaluation methodology already used for pedestrian detection, to obtain realistic figures of the capabilities of a hand posture detection and classification method.

With respect to the hand recognition problem, there are in the literature two main approaches: the methods based on wearable devices and non-intrusive methods. The methods based on wearable devices are methods where the user needs to use sensing gloves [6], markers (e.g., LED’s) or any other type of device to facilitate the process of hand recognition. The non-intrusive techniques are based on the data provided by depth sensors and/or camera sensors. In our work, we focus on the non-intrusive methods.

In the non-intrusive methods, a common strategy is to use the depth information or the image information to isolate the hand from the background [7]. After the hand isolating segmentation process, a classification process is performed [8]. There are several works in the field that apply these two steps to recognize hand gestures using depth information [9,10,11,12] and using the image information [13]. In these methods, if the segmentation process exhibits a poor performance, the recognition system decreases its accuracy dramatically. Some depth sensors are not designed for outdoor applications, and the use of these devices requires an external power source. In the same way, the computer vision methods that use the image information to perform the hand segmentation have problems with different lighting conditions or complex scenarios.

Other types of approaches to recognize hand gestures using computer vision-based methods are reviewed by Murthy and Jadon [14]. In recent works, the hand recognition systems proposed by Pisharady *et al.* [15] and Li and Wachs [16] achieve high accuracy rates (93% and 98%, respectively), but the time needed to classify an image is impractical for an HRI task. According to Rautaray and Agrawal [17], achieving performance near to real time is an important feature for hand recognition systems. However, there is no standard hand posture dataset to perform a fair comparison between different methods. The lack of a standard hand posture dataset implies that there is no common methodology to perform the evaluation of the system.

To avoid the problems with hand segmentation and the time needed to process an image, the object detection methods based on boosting have proven to exhibit a good compromise between accuracy and time. The object detection framework first proposed by Viola and Jones [18] is an example of such a compromise. The Viola–Jones framework has been the base for several approaches to detect hand postures [19,20,21] and hand gestures [3]. In all of these works, the authors use the same image dataset. One problem with the Viola–Jones framework is the low accuracy to detect objects with mobile parts, for example pedestrians. Dalal and Trigs’ [22] framework for pedestrian detection overcame this problem by using the histogram of oriented gradients and support vector machines. Several works in the pedestrian detection area have been proposed [23], but these approaches need extra hardware (GPU) to be executed in real time.

From this review, we observe the need for hand recognition systems capable of recognizing the hand gestures in the least possible time. For these reasons, we propose the use of the RTDD originally proposed by Ali *et al.* [24] to implement a human-robot interface based on hand gestures. Using the RTDD as the core detector, we train a set of detectors specialized in a single hand posture and use them jointly to perform multiclass hand posture detection. The parallel operation of the detectors is accelerated by sharing the computation of features among all of the sets of RTDDs.

Furthermore, we observe the lack of a standard dataset and a standard evaluation protocol. A good evaluation protocol gives meaningful information about the performance of the system. That is why we introduce the use of the evaluation methodology proposed by Dollár *et al.* [23,25] for pedestrian detection in the hand posture recognition problem. Using the evaluation protocol proposed by Dollár, it is possible to achieve a fair comparison with other proposed methodologies. The proposed vision system can interface with the ROS (Robot Operating System) to provide information and perform the HRI.

This paper is organized as follows. First, we review the hand posture detection methods based on boosting, the available hand posture datasets and the evaluation methodology in Section 2. Our proposed methodology is described in Section 3, where we describe the RTDD and the modifications proposed to perform multiclass detection. The results obtained using the proposed methodology are shown in Section 4. In Section 5, the conclusions of this work are given.

## 2. Hand Gesture Recognition

Many methods have been proposed to solve the hand gesture recognition problem. Hand gesture recognition is divided into three main phases: detection, tracking and recognition. For an extensive revision of the proposed methods in the literature, the reader can review [4]. In our review, we focus only on the hand detection step.

First, we describe the methodologies to perform the hand gesture recognition using computer vision and how the performance evaluation is carried out. We also review the main issues of the learning process to obtain a good classifier. Finally, we address the main characteristics of the hand gesture database to help the system be useful in a more realistic scenario.

### 2.1. Hand Gesture Recognition

Hand gesture recognition involves a sequence of hand postures in a short time span and the classification of this sequence. A hand posture is a static configuration of the hand [19]. There are many works focused on the detection of hand postures, because this is the base step to recognize gestures.

Hand detection is the task of obtaining a bounding box (BB) where the hand is located in the image plane. To perform the hand detection, the methods need to deal with several challenging scenarios, such as:

- Lighting variations [26].
- Hand segmentation [27].
- Different hand morphologies [28].
- Signal noise [29].

### 2.2. Learning Using a Boosting Approach

The detection methods based on boosting approaches (e.g., those that use the AdaBoost algorithm) have shown a good performance to address the aforementioned problems. The AdaBoost algorithm is an ensemble of weak classifiers (often called weak learners) that are combined to obtain a strong classifier. In object detection frameworks, a weak classifier is defined by a set of features and a classifier method. The features are selected during the training step. The weak classifier allows one to solve some problems, like lighting variations and signal noise. Because the AdaBoost algorithm learns from examples, it is necessary to have a good database. For the hand recognition problem, a good database must contain several hand morphologies and different skin colors, and the hand postures must appear in simple and complex backgrounds. As the number of good examples in the database increases, the generalization of the system also increases.

The first approach to recognize hand gestures using boosting was proposed by Chen *et al.* [19]. In their work, the authors proposed the use of the Viola–Jones framework for hand posture detection, and the different hand postures are used as a grammar. In order to discard false positive detections, Cao *et al.* [30] use a skin color model jointly with the Viola–Jones detector. If the number of pixels in the detected area is lower than a threshold, the area is discarded. Another approach uses the skin color information present in the image, as in the work proposed by Tran *et al.* [21].

Additionally, there are works that use the AdaBoost learning algorithm with other base features. Yao and Li [31] propose the use of the key points given by the SURF (speeded-up robust features) algorithm as the features of a weak classifier. During the boosting procedure, the combination of an interest point and a threshold that minimizes the error in classification is selected. This procedure is repeated until a minimum error is reached. The use of the key points as a feature for the weak learner is also proposed by Wang and Wang [32]. Instead of using the SURF algorithm to obtain the key points, they use the scale-invariant feature transform (SIFT).

### 2.3. Hand Posture Dataset

The human-computer interface (HCI) community has accepted several datasets as benchmarks for the hand gesture recognition problem. Each dataset provides its own information: motion information, color information, depth information, annotations, *etc*.

We summarize the characteristics required for a good hand posture dataset:

The variety of backgrounds in the dataset must cover scenarios going from simple to complex. The background complexity may have a big impact on the detection methods according to the technique used. A challenging scenario for the detection methods that are based on color segmentation is when the background is similar to the skin tone. The outdoor and indoor scenarios provide different backgrounds and changes in the illumination. These types of scenarios are also a good addition to the dataset.

The number of hand postures in the dataset is an important feature, as is the number of samples per hand posture. In most cases, the number of hand postures in a dataset is based on the needs of the authors, and it has a strong relationship with the target application. The number of samples in a dataset is important, because each sample is an instance of the execution of the hand posture. The user does not perform the exact gesture in each execution, and with that, the learning method increases its generalization. The dataset also should include hand postures under different geometric transformations (e.g., rotation, translation, scale).

The dataset provides additional information, like the hand segmentation regions, the interest points or some other type of relevant information for each image. This information is useful in the learning step and also to compare the performance of several methods. The last important feature in a dataset is to include the hands from different persons, ethnicities and hand morphologies.

In the next paragraphs, we review the characteristics of some hand posture datasets. We only focus on the datasets available to download. The dataset proposed by Liu [33] is focused on depth and color information. The Kinect sensor was used to acquire the depth and color information of the hand gestures of six individuals. These types of datasets are important when the depth information is important in the recognition process.

The dataset proposed by Kim *et al.* [34,35] is composed of several image sequences. The images were taken at different lighting conditions, and all images are of the back of the hand. This dataset is focused on motion information. There are three canonical hands in this dataset. With this small number of hands, it is not possible to cover several hand morphologies.

**Figure 1:**
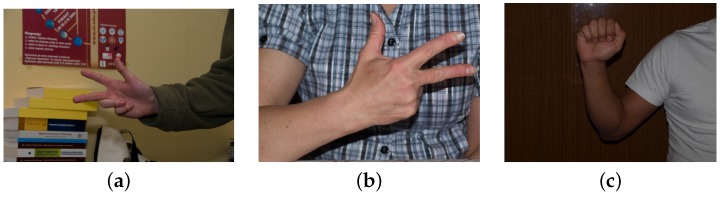
Samples of images from the dataset HGR (Hand Gesture Recognition) [36].

Information about the skin segmentation and the hand key points is provided by Kawulok *et al.* [36]. The dataset was built from 18 individuals. The images were shot under different lighting conditions, and the backgrounds are simple in most of the cases. In Figure 1, some images from this dataset are presented. There are different lighting conditions, but the background in the images is only from indoor scenes, and in most of the cases, the background is homogeneous, as for example in the Figure 1c. Because of the lack of challenging scenarios, this dataset is not the most suitable for hand posture recognition.

Triesch *et al.* [37] proposed a dataset focused on simple and complex backgrounds. The background complexity is only from indoor environments, and in most of the cases, there are no significant lighting variations.

The National University of Singapore (NUS) Hand Posture Dataset II [15] (available at http://www.ece.nus.edu.sg/stfpage/elepv/NUS-HandSet/.) fulfills most of the requirements of a good image dataset. The postures were taken from individuals from different ethnicities. There are different shapes, sizes and skin color in the hand postures. This dataset also adds cases where the hands appear among humans in the background. None of the datasets reviewed have these types of cases. The number of classes presented in the dataset is 10. The first version of the NUS hand posture dataset was proposed by Kumar *et al.* [38].

The NUS Hand Posture Dataset II is composed by 2750 images that contain a hand posture and 2000 background images (without any hand posture). The 2750 images are divided in two subsets, one with 2000 images and one with 750 images.

The first subset is composed of images with complex backgrounds and different lighting conditions. The images were take from 40 individuals (five images per class and per individual). The image size is 160×120. We refer to this dataset as NUS-II-A.In the second subset, human activity is added to the images. The images have the same complexity in the backgrounds and lighting condition. In this subset, 15 individuals were used to shoot the images. The image size is 320×240. We refer to this dataset as NUS-II-B.

**Figure 2:**
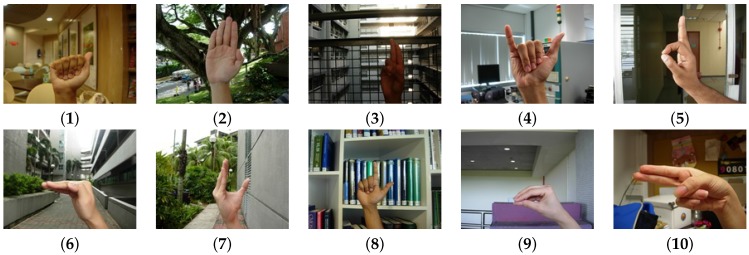
Samples of each hand posture (including its number labelling) that are contained in the National University of Singapore (NUS) Hand Posture Dataset II. The samples show the complexity of the backgrounds, the different lighting conditions and several hand morphologies.

In Figure 2, some samples taken from the dataset are presented. In these samples, the hands are in indoor and outdoor scenarios; this introduces different lighting conditions. Furthermore, we can see several hand morphologies and skin colors.

### 2.4. Evaluation Methodology

The evaluation methodology serves to measure the performance of a system under different conditions. There are many evaluation protocols and different datasets in the object detection and recognition area. This implies that in most of the cases, we cannot achieve a direct comparison among the different approaches.

The evaluation of the detection systems can be complex and tricky, as Dollár *et al.* explain in their work [23]. There is no standard evaluation protocol to evaluate a system where the hand posture detection is involved. This lack of standardization is in part due to the use of different datasets. The datasets provide the samples in two types: the object is the whole image or there are several objects in the image. The Caltech dataset 101 [39] is composed of images where the object is the whole image. In contrast, the Pascal VOC (Visual Object Classes) 2007 datasets [40] provide images with several objects per image and also provide annotations about the position and the classes of the objects.

The other part of the problem is how the performance of the system is evaluated. The approach most used for evaluation purposes is the per-window (PW) evaluation. The PW evaluation is performed by cropping the positive and negative samples and then using a classification accuracy measure. The problem with this type of evaluation is the weak correlation between the performance of PW evaluation and the performance of the detector over the full image. The PW evaluation is used in several works [19,30,41].

In the work of Pisharady *et al.* [15], the evaluation of the system is done by taking into account if the hand is detected or not. They present their results using a receiver operating characteristic (ROC) curve. The curve is obtained by varying a probability threshold and calculating the false positive rate and the true positive rate with the new configuration. They use the NUS hand posture dataset to perform all of the tests. The ROC curve is a common way to present the performance of the system under different conditions.

The object detection frameworks are described by several parameters that can be directly compared. For example, there are hand posture detectors that use histogram of oriented gradient, Haar-like features or variance as base features and others that use color segmentation as an input, *etc*.

## 3. Methodology

We propose a multi-class hand posture detector that uses a specialized RTDD for each hand posture. First, we describe the methodology for designing each of the RTDDs; secondly, we describe how they are organized in order to speed up computations by sharing some elements. A description of the learning step for the RTDDs is also included. The weighted sampling method is used for the training phase of the RTDDs. The proposed HRI system can handle four hand postures simultaneously. The performance of the entire HRI system is computed using a full image evaluation method. This method is also described below.

Given a test image, we generate 8 scaled copies of each edge map. For all of them, the edge map is computed, and from it, eight rotated images are generated. For each of the eight scales, a scanning window of 50×50 pixels is displaced through the entire image. In the scanning window, all of the weak classifiers belonging to each of the RTDDs are tested for matching. As a result, we obtain a list of candidate positions and scales for each hand posture recognized by the proposed system.

For each hand posture, we run a non-maxima suppression step to decide the final position of the detected instances of the postures.

### 3.1. Real-Time Deformable Detector

For the sake of completeness, we are going to describe the RTDD. The RTDD is the result of the application of the AdaBoost learning process to an image dataset containing positive and negative samples of the hand posture to be recognized. It tries to find an optimal pose-indexed feature for each weak classifier included in the RTDD and the optimal region to compute the dominant orientation to compute such a pose-indexed feature. That is achieved by performing a search in the problem space to reduce the weighted error of the image samples. A detailed description of this procedure is given in the following paragraphs.

A pose-indexed feature is a parameterized feature that contains information about the pose, e.g., in-plane rotations, out-of-plane rotation, position, deformation, *etc*. This information is useful to process the signal in an efficient way. In order to use the pose-indexed feature to train a system, we need a dataset annotated with all information required by the pose-indexed feature. The annotation process is performed by humans, even if they can introduce errors during this process.

To avoid the dataset annotation process, Ali *et al.* [24] propose the use of a pose estimator jointly with the pose-indexed feature. The pose estimator gives the necessary information to the pose-indexed feature. The AdaBoost procedure allows one to choose the best combination of the pose-indexed feature and pose estimator. In each step of the learning process, a search for the best combination of these two elements is performed.

The base feature used by the pose-indexed feature is the combination of two ideas: the search of features used in the Viola and Jones framework [18] and the histogram of oriented gradients proposed by Dalal and Trigs [22].

The features are the elements of a histogram of gradient orientations that is derived from the edges of the image. The edge orientations are quantized in *q* bins. Set *ϕ* as the possible edge orientations in the range Φ=[−π,π], the quantized bin as $\hat{\Phi }=\left\{0,\frac{2\pi }{q},\frac{4\pi }{q},\ldots ,\left(q-1\right)\frac{2\pi }{q}\right\}$. Then, $\forall e\in \hat{\Phi }$, x∈I and l∈{1,…,W}×{1,…,H}:

(1) $$\xi_{e}\left(x,l\right)\in \left\{0,1\right\}$$

**Figure 3:**
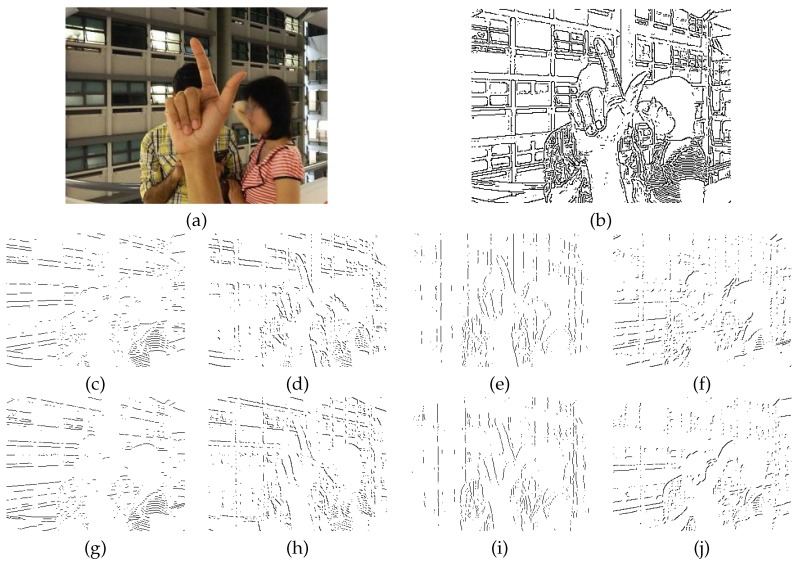
Edge maps for eight orientations: (**a**) input image taken from the NUS-II image dataset and (**b**) edges calculated from the input image. Integral images are computed from this edge map. Their rotated versions are used to compute the pose-indexed feature.

Equation (1) is used to denote the presence of an edge pixel in a quantized orientation *e* at pixel *l* in image *x*. There are different computer vision methods to obtain the edges of an image. In this work, we use the edge detector proposed by Fleuret *et al.* [42], because of its low computational cost. In Figure 3, we show the results of this edge detector applied to an image from the NUS-II image dataset. With this edge detector, we use q=8.

The base feature is then defined by a subwindow *R* inside the region r×r in the image plane (Equation (2)). The base feature is defined as the sum of all edge pixels inside the subwindow *R* with an orientation *e* over the sum of all edge pixels inside the subwindow.

(2) $$h_{R,e}\left(x\right)=\frac{\sum_{m\in R}\xi_{e}\left(x,m\right)}{\sum_{d\in \hat{\Phi },m\in R}\xi_{d}\left(x,m\right)}$$

The computation of the base feature is performed by using *q* integral images computed from the edge maps images, one integral image for each edge map. Integral images are a fast way to compute the area of a rectangular region. That allows to compute the features in constant time. The first work that used integral images was the one proposed by Viola and Jones [18]. The pose-indexed image feature is defined from the base feature. First, we define the image plane as $\Theta_{1}=\left\{1,\ldots ,W\right\}\times \left\{1,\ldots ,H\right\}$ and an orientation as $\Theta_{2}=\left[-\pi ,\pi \right]$. For a rectangular subwindow *R* and poses $l=\left(u,v\right)\in \Theta_{1}$ and $\theta_{2}\in \Theta_{2}$,

(3) $$R_{l,\theta_{2}}$$

defines a rectangular window in the image plane, rotated by an angle $\theta_{2}$ and translated to (u,v). In the same way, we define $e_{\theta_{2}}$ as the new orientation resulting from the rotation by $\theta_{2}$ of the edge orientation *e*. With $R_{l,\theta_{2}}$ and $e_{\theta_{2}}$, we can redefine the base feature $h_{R,e}$ as a pose-indexed feature as:

(4) $$g_{R,e}\left(\left(l,\theta_{2}\right),x\right)=h_{R_{l,\theta_{2}},e_{\theta_{2}}}$$

The pose-indexed feature is computed by using 2q integral images, *q* integral images for the edge map and *q* for each rotated map by π/4. In our setup, we set q=8, which results in 16 integral images to compute the pose-indexed feature. The next step is the definition of the pose estimator.

The pose estimator is used in the evaluation of the pose-indexed feature. The pose estimator is used to obtain a dominant orientation in $\theta_{2}\in \Theta_{2}$ from a region Λ in the location l=(u,v). It is defined as:

(5) $$\eta_{\Lambda }\left(l\right)=\underset{e\in \hat{\Phi }}{arg max}h_{\Lambda_{l},e}$$

**Figure 4:**
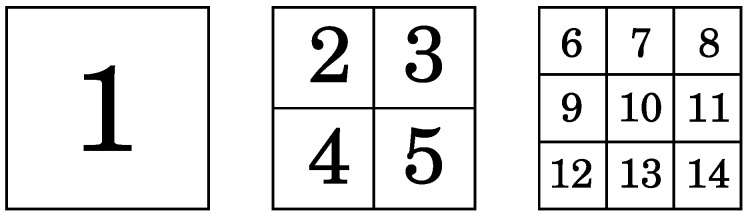
Pose estimators used to compute the pose-indexed feature. The pose estimators are calculated in the sliding windows r×r in different positions and scales.

In our setup, we use 14 pose estimators. Figure 4 shows the shape of the pose estimators. There is no need for extra processing, because pose estimators are computed using the same integral images used by the pose-indexed feature.

The learning setup is based on the AdaBoost learning algorithm. The first step is the initialization of the weights. The number of negative samples is based on the number of positive samples *a*. The *J* parameter defines the number of weak learners that are tested in each round. The weak learners are created randomly in each step to reduce the learning time.


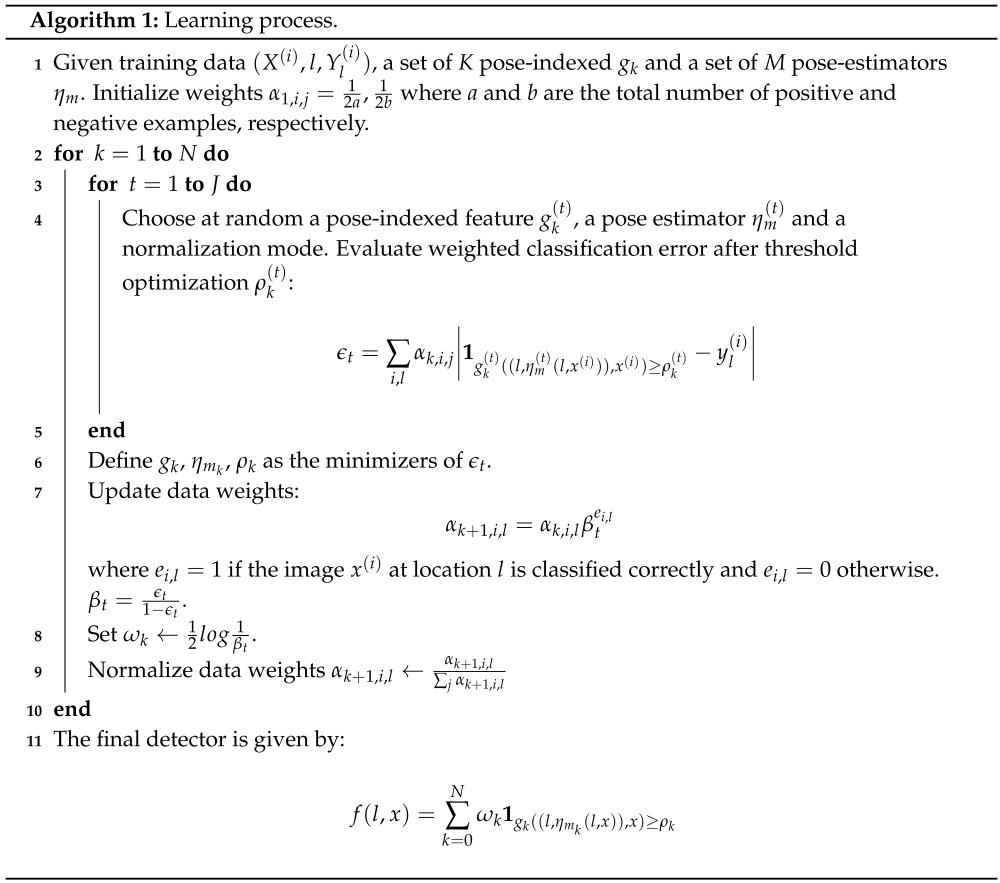


The proportion between the negative samples and the positive samples is based on the complexity of the pattern to learn. The proportion proposed by Ali *et al.* [24] is b=Ca with C=10. In our experiments, we use C=20. This choice results in a good performance whilst reducing the training time needed. A larger value of *C* will imply the need for more computational resources and time to perform the learning step. **Algorithm 2:** Detection Outline**1** Given a patch from image *x* and location *l*, **2** evaluate all *M* pose estimators $\eta_{m}$. **3** Evaluate the strong classifier: $$f\left(l,x\right)=\sum_{k=0}^{N}\omega_{k}1_{g_{k}\left(\left(l,\eta_{m_{k}}\left(l,x\right)\right),x\right)\geq \rho_{k}}$$

### 3.2. Learning from Examples

The learning algorithm learns the objects from the examples given during the training phase. In most cases, these samples are obtained from standard datasets (pools).

The quality of the samples used during the learning has a significant impact on the resulting classifier. For a binary classifier, the samples are divided into positive and negative ones. The positive samples are those containing the pattern that we want to learn. The negative samples are those containing anything else different from the positive samples.

Some image datasets have a large amount of samples that make it impractical to use all positive and negative samples during the learning step [43]. However, we need a large number of positive and negative images to learn the desired pattern. This implies the use of a huge amount of computer resources and a long time consumed in the learning step. We have limited computer resources to handle a huge amount of samples. Furthermore, the time needed to learn from a huge image dataset is too long. Furthermore, the learning algorithm needs good quality positive and negative samples to effectively learn the desired pattern.

For these reasons, some strategies were developed to use the image samples in an efficient way. These strategies are focused on choosing samples from the entire image dataset to build a small subset. Then, this small dataset is used in the learning process.

Some common strategies to perform the sampling in the boosting methods are: trimming [44], where the samples under a certain threshold are discarded, unique uniform sampling (UUS), where all samples have the same weight, and weighting-by-sampling, proposed by Fleuret and Geman [42].

We want to train an ensemble that uses a minimum number of weak classifiers. The weak classifier used by the RTDD is a decision stump. The computational cost is reduced by using fewer decision stumps. For this reason, we combine two methodologies in the learning step.

The first methodology is focused on getting good negative examples from the entire pool. The original pool was constructed with outdoor/indoor images, with images that contain text and texture images. The samples are selected using a classifier trained with random samples and with a small number of weak classifiers. This idea is similar to the work proposed by Malisiewicz *et al.* [45].

The second methodology used by the RTDD is the weighted sampling [42]. This method proposes the replacement of negative samples for new negative examples according to the weights during the training. The method splits the number of *V* decision stumps, used in *B* blocks of *U* decision stumps. At the start of each block, the sampling and replacement process is performed. This procedure helps to remove negative samples that had been classified incorrectly during the learning process.

These methodologies allow us to reduce the number of decision stumps needed to classify a hand posture. The positive samples are obtained from a manually annotated dataset, and the negative samples are obtained from a different dataset.

### 3.3. Full Image Evaluation

The evaluation methodology used in this work is the one proposed by Dollár *et al.* [23]. In the following, we describe this evaluation methodology.

To perform the full image evaluation in a single frame, we need a ground truth bounding box ($BB_{gt}$) and a detected bounding box ($BB_{dt}$). Each $BB_{dt}$ has a score or confidence. If the image has several objects, we need a $BB_{gt}$ for each object. A BB is defined by the position (u,v), the width *w* and the height *h*.

A $BB_{dt}$ and a $BB_{gt}$ are the same object only if the overlap between these bounding boxes exceeds 50% (see Equation (6)). The overlap threshold at 50% has been analyzed for several tests. If the overlap threshold is increased, the test performance decreases, because the $BB_{dt}$ must be more accurate in position and size with respect to the $BB_{gt}$. Otherwise, if the overlap threshold is decreased, the test performance increases a little.

(6) $$a_{o}=\frac{area(BB_{dt}\cap BB_{gt})}{area(BB_{dt}\cup BB_{gt})}>0.5$$

For each $BB_{dt}$, we look for a matching with the $BB_{gt}$. When one $BB_{dt}$ and one $BB_{gt}$ satisfy the overlap condition, it counts as a true positive. When two or more detections satisfy the overlap condition for a $BB_{gt}$, the detection with the highest value is taken as the detection for the $BB_{gt}$. This is useful when there are two or more objects that are close together. If a $BB_{dt}$ does not have any matching, it counts as a false positive. Otherwise, if a $BB_{gt}$ does not have any matching, it counts as a false negative.

Using the full image evaluation, we can plot the performance of the detector under different configurations. To build the graph, we evaluate the RTDD varying the threshold of the detector and evaluating the detector. Using this evaluation, we can also know the best configuration of the detector.

**Figure 5:**
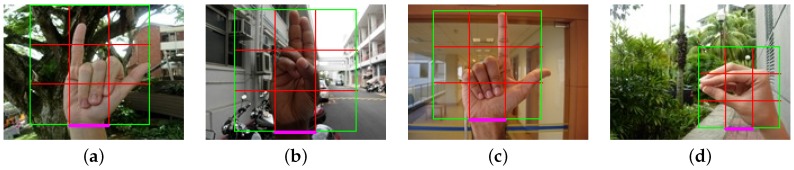
Examples of annotations in the dataset.

In order to perform the full image evaluation, we annotate the entire dataset (NUS-II dataset) with a square window. The rectangular shape fits better for some hand postures, but we decided to take a square window to handle different hand postures in the same window. In Figure 5, we show some examples of the annotation using images form the NUS-II dataset. We use the wrist as a reference to set the square window (Figure 5a–c). In some cases, the wrist is not useful to annotate the hand. For example, in Figure 5d, we use the center of the hand as a reference. They are images where the square annotation can be centered at the hand posture. In these cases, we cover all of the hand posture. We provide the image annotations for the NUS-II image dataset as additional material of this work for comparison purposes.

## 4. Results

The results obtained from the proposed methodology are presented in this section. We describe the experimental setup used in the learning and testing steps. We divide the experiments into two sections.

In the first section, we show the results obtained in the detection of one hand posture. These results are obtained using the full image evaluation approach. Also in this section, we show some examples of the hand posture detection experiments. In the second section, the results obtained from the human-robot interface (multiclass hand posture detection) are presented. We use four gestures to build the interface. We show a confusion matrix to show the results.

In the learning and testing steps, we use the NUS-II-A dataset. Furthermore, we use images from other datasets (e.g., VisTeX (Vision Texture) , Brodatz, Caltech 101, among others) to increase the number of negative images.

The learning process is as follows. First, we generate a pool of negative samples. We train a detector with a small set of positive and negative samples to perform that. Using this detector, we generate a pool of 1,000,000 negative samples. Not all of the images in the pool are used during the training step. The number of used negative samples is based on the number of blocks *B*; in our setup, around 100,000 samples are used from this pool.

The negative images used to perform this procedure are the background images provided by the NUS-II dataset and the images from the others datasets. In the negative pool, there are a few samples that are difficult to learn during the training process. This is not a problem, because the weighted sampling strategy used by the RTDD discards these samples during the re-sampling procedure.

**Figure 6:**
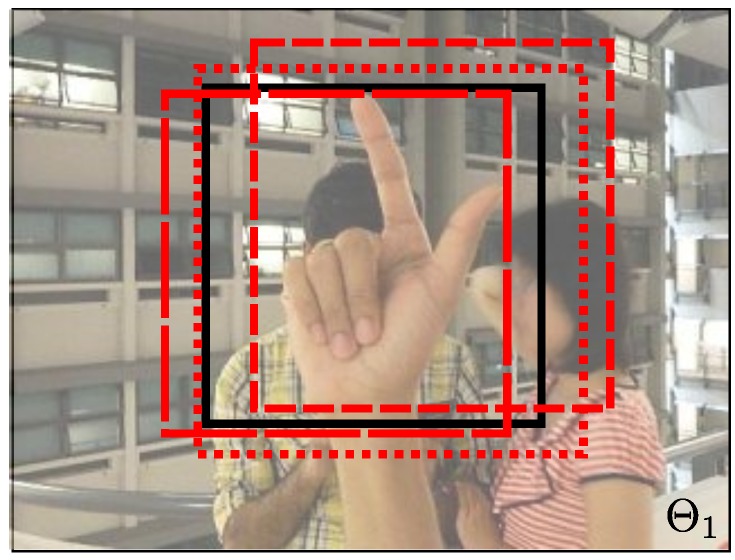
Positive sample generation. At the center of the image, we have the $BB_{gt}$, and the dashed windows are the positive sampled BB. We generate samples by perturbing the position and the size of the $BB_{gt}$ using Gaussian noise.

The pool of positive samples is created by randomly perturbing the set of $BB_{gt}$. We add Gaussian noise $N(\mu ,\;\sigma^{2})$ to the $BB_{gt}$. The result is a change in the size of the bounding boxes and a random displacement of the $BB_{gt}$. Figure 6 shows an example of this procedure. The $BB_{gt}$ at the center is the reference, and the dashed $BB_{gt}$ represents some of the generated samples.

In all tests, we only generate a number of positive images that are a multiple of 200 (the number of samples per class in the NUS-II-A dataset). We use the NUS-II-B dataset for testing purposes. The number of images in the NUS-II-B is 75, and we use these images without any perturbation.

Once the training samples have been generated, the next step is to train the RTDD. We use 1000 positive samples and 20,000 negative samples obtained from the pool, to train the detector. We configure the learning setup with V=500 decision stumps, used in B=5 blocks of U=100 decision stumps.

We select this setup to find an efficient detector with a small number of decision stumps. When the number of decision stumps increases, the time needed to perform the detection also increases. The AdaBoost performs a search for the best decision stump at each iteration. The number of examined features at each iteration was 1000. This value is that proposed by the authors for RTDD in [24]. The threshold was computed using an exhaustive search procedure. Particularly, we use the method proposed by Wang [46] to calculate the threshold.

Next, we are going to show the results obtained over the hand postures in the NUS-II-A and NUS-II-B; first, the hand posture detection and then the multiclass hand posture detection. In all of the experimentation, we use a general-purpose computer with 8 GB RAM and a processor running at 2.7 GHz.

### 4.1. Hand Posture Detection

In this part, we perform individually the full image evaluation for each hand posture in the dataset NUS-II. These results allow us to find the best configuration of the detector. For all tests, we use a classifier window size of 50×50 pixels. The classifier scanned the images using a step of five pixels.

**Figure 7:**
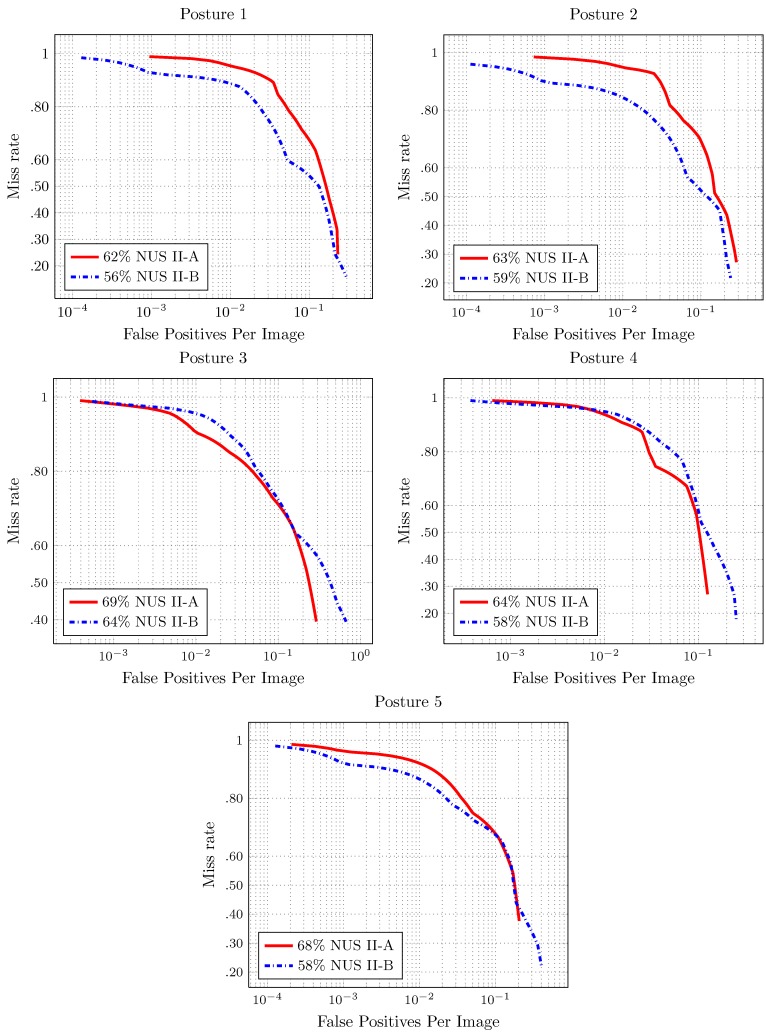
Results of the full image evaluation for Hand Postures 1–5 in Figure 2. The score is the log average of the miss rate.

**Figure 8:**
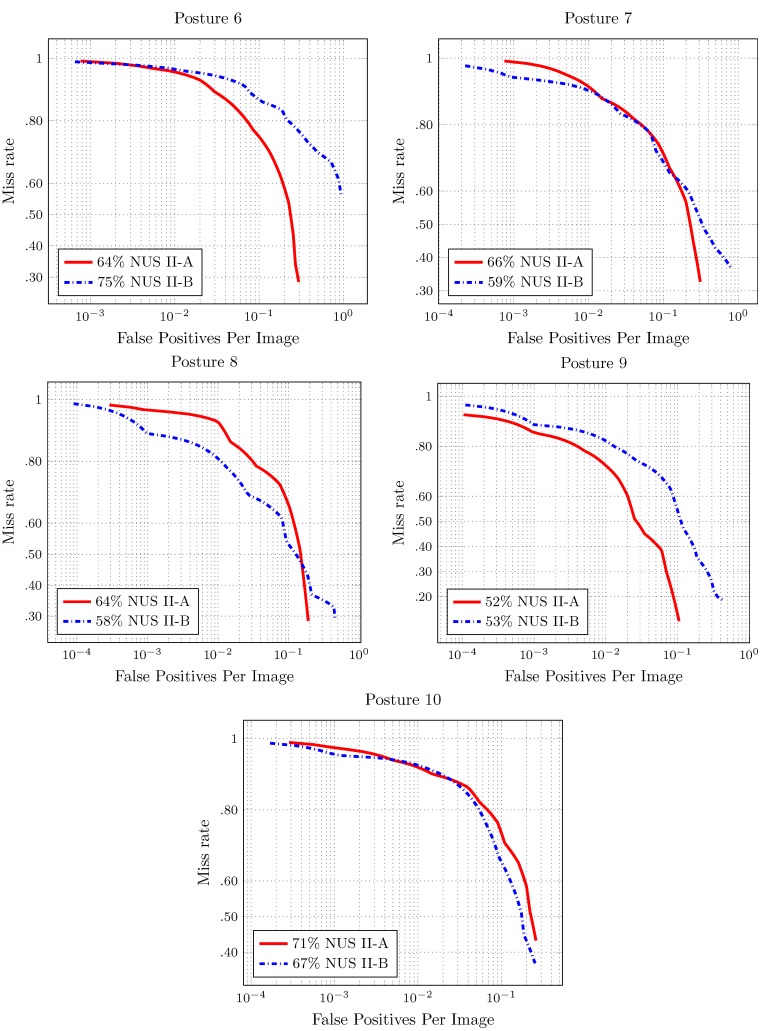
Results of the full image evaluation for Hand Postures 6–10 in Figure 2. The score is the log average of the miss rate.

The results for the full image evaluation are shown in Figure 7 and Figure 8. The score shown in the results is the log-average miss rate. This score is a common reference to summarize the detector performance.

The detector performance is better when the area under the curve is smaller. In the full image evaluation results, the dataset has a significant impact on the performance of the detectors. This behavior is similar to that presented for the pedestrian detection evaluation over the different datasets [23].

In most of the cases, there is good performance in the number of false positives per image. The exception is Hand Posture 6, in NUS-II-B. In this evaluation, the number of false positives is more than the number of images (75). Furthermore, the value of the miss rate is high for this hand posture. That is because the individuals in the dataset perform the hand posture in different ways.

### 4.2. Multiclass Hand Posture Detection

In order to build a multiclass hand posture detection system, we chose a subset of hand posture numbers. All of the postures are referenced to Figure 2. Posture 6 is discarded because, given that users perform it in different ways, the performance evaluation of its detection is poor (see Figure 8). To perform Hand Postures 4, 5, 7, 9 and 10, we need to make a twist of the wrist or move the arm. These hand postures are also discarded.

We use Hand Postures 1, 2, 3 and 8 to perform the multiclass hand posture detection. These hand postures show a good performance in the NUS-II-A and NUS-II-B datasets. Each point in the resulting graph is a configuration of the detector. To choose the best configuration, we use the F-measure. We use the detector at the same time over the entire image. When two or more detections are true in the same place, we select the detection with a higher weight.

The system evaluation was performed in two parts: per window evaluation and full image evaluation. We use the NUS-II-B dataset to evaluate the performance of the system.

The results are presented in Table 1. We can see good performance of the system with an accuracy of 90.66% in the per window evaluation test. In the full image evaluation, we have 75.33% accuracy. This decrease in performance of the system is expected given the results obtained in full image evaluation works [23]. The difference in performance between the per window evaluation and full image evaluation is because of the different elements that are evaluated in each test. To perform the per window evaluation, the samples are scaled to the size of the classifier. Moreover, the full image evaluation measures the performance of the whole detection system. The position and size of the detection are not taken into account in the per window evaluation.

**Table 1:** Confusion matrix for the four poses selected using the NUS-II-B. (**a**) For the confusion matrix for the per window evaluation in the NUS-II-B, the accuracy is 90.66%; (**b**) results for the full image evaluation over the NUS-II-B. The accuracy is 75.33%.

(**a**) Sample evaluation									(**b**) Full image evaluation					
	**1**	**2**	**3**	**8**	**Accuracy**					**1**	**2**	**3**	**8**	**Accuracy**
**1**	69	0	0	6	92.00%				**1**	55	4	3	12	73.33%
**2**	4	62	4	5	82.66%				**2**	2	65	1	2	86.66%
**3**	1	0	71	3	94.66%				**3**	3	14	44	11	58.66%
**8**	5	0	0	70	93.33%				**8**	11	4	3	62	82.66%
				Total	**90.66**%								Total	**75.33**%

One advantage of using the RTDD is the lower time needed to process an image in comparison with the method proposed by Pisharady *et al.* [15] that needs 2.65 s to process an image of a size of 320 pixels × 240 pixels. The methodology proposed by Pisharady needs first to create a saliency map and then extract the hand from this map. This is a time-consuming process and the main reason for the low time performance of this method. Another result over an early version of the NUS hand dataset achieves an accuracy of 95% [38]. This result is obtained by classifying the whole image as an input, in contrast to the full image evaluation used in our work. Furthermore, the time needed to perform the feature extraction is around 1.3 s. This makes the algorithm impractical to implement an HRI.

In Table 2, the processing time per frame is shown. The system can perform the detection step at a frame rate close to 10 frames per second.

**Figure 9:**
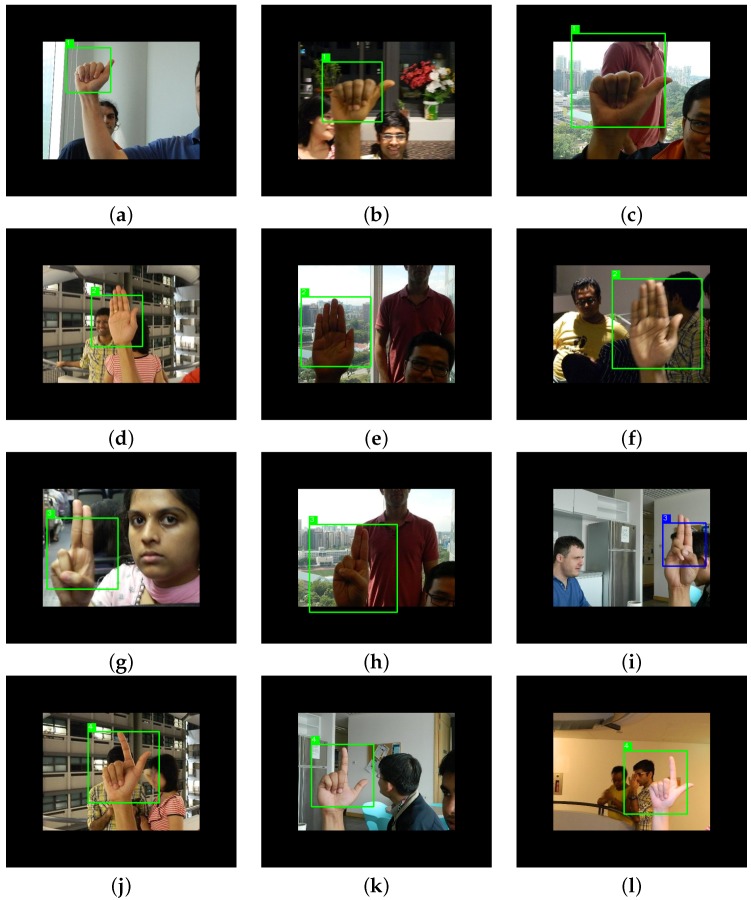
Results obtained from the multi-posture detector in the NUS-II-B. We select images with different lighting conditions, different backgrounds and different users. In (**i**), the detection failed.

We show some qualitative results of the detection system in Figure 9. The black pixels around the image are added to enable the detection of the hand postures at the borders of the image. The detection in Figure 9i fails because the overlap is less than 50%, even though the class of the detection is correct.

**Table 2:** Time performance of the system. The multi-posture detector uses 1550 stumps.

**Image Dimension**	**Frame Processing Time (ms)**	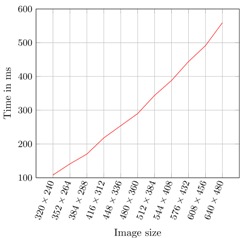
320 × 240	108	
352 × 264	141	
384 × 288	170	
416 × 312	218	
448 × 336	254	
480 × 360	290	
512 × 384	344	
544 × 408	388	
576 × 432	444	
608 × 456	491	
640 × 480	559	

## 5. Conclusions

We have presented a multiclass hand posture recognition system based exclusively on computer vision. We have also shown the main issues arising in the hand posture detection problem and how they can be overcome with the proposed system. The developed system uses an ensemble of real-time deformable detectors to handle the detection of multiple classes of hand postures.

Given that there is not yet a standard methodology to test the hand posture detection framework, we propose the use of an evaluation methodology that is already used to evaluate pedestrian detectors. This methodology enables one to have a fair evaluation of a detector, instead of using the per window evaluation procedure.

The dataset used to learn an object is a crucial decision. For this reason, we provide a review of the available hand posture datasets and what the features needed by a dataset are. We select hand postures from the used dataset that are easy to perform. These hand postures also exhibited a good performance in the full image evaluation.

Our main contributions are the design of a multiclass hand posture detector, the evaluation methodology and the use of a complex database in the training step. The multiclass hand posture detector performance with respect to time is better than other similar approaches.

The GPU parallelization of the learning algorithm is one of the ideas to improve this work. The time to train a RTDD is around 6–8 h. This time is still huge, if we want to make more experimentations with different configurations of the size of the window, other pose estimators and others elements of the method.
